# A new treatment for reliable functional and esthetic outcome after local facial flap reconstruction: a transparent polycarbonate facial mask with silicone sheeting

**DOI:** 10.1007/s00238-017-1306-y

**Published:** 2017-05-31

**Authors:** Sander B. Kant, Patrick I. Ferdinandus, Eric Van den Kerckhove, Carlo Colla, René R. W. J. Van der Hulst, Andrzej A. Piatkowski de Grzymala, Stefania M. H. Tuinder

**Affiliations:** 1 Department of Plastic Surgery, Maastricht University Medical Center, P Debyelaan 25, 6229HX Maastricht, The Netherlands; 2 KU Leuven, Department of Rehabilitation Sciences, Faber, Universitaire Ziekenhuizen Leuven, Leuven, Belgium; 3 Department of Physical Medicine and Rehabilitation and Burns Center, Universitaire Ziekenhuizen Leuven, Leuven, Belgium

**Keywords:** Facial flap reconstruction, Pressure mask, Silicones

## Abstract

**Background:**

Facial flap surgery predominantly leads to good functional results. However, in some cases, it can cause unsatisfactory esthetic results. They include persistent erythema, pincushioning, and development of hypertrophic scars. Conservative, reliable treatment for facial flaps is lacking. Pressure and silicone therapy have proven to result in significant improvement in scar erythema, pliability, and thickness in postburn hypertrophic scars. By combining these therapies in a facial mask, the esthetic outcome of facial flaps could be improved. In this retrospective study, the efficacy of a unique transparent face mask containing silicone sheets on the esthetic outcome of postsurgical facial flaps is assessed.

**Methods:**

Twenty-one patients were assigned to facial pressure mask therapy after they underwent facial flap surgery between July 2012 and September 2015. Patients were treated for a mean duration of 46 weeks. The effects of pressure mask therapy were examined by means of the Patient and Observer Scar Assessment Scale (POSAS).

**Results:**

All POSAS components showed a reduction between start and end of therapy, while itchiness, pigmentation, pliability, thickness, and relief of the flap improved significantly (*P* < 0.05). Mean total and patient score showed significant reduction between start and end of therapy.

**Conclusions:**

This study shows that a facial pressure mask layered with silicone results in noticeable flap improvement with a long-lasting result.

Level of Evidence: Level III, therapeutic study.

## Introduction

Worldwide, the number of people that suffer from skin cancer is increasing every year. Surgical resection is the standard of care in facial plastic surgery [1]. One of the standard procedures to close facial defects is local or regional soft tissue flaps [2].

In order to cover a defect and to restore facial anatomy as well as possible, many options for surgical flaps exist. Well-known and commonly used flaps include Abbe, rhomboid, forehead, bilobed, and glabella flap [3, 4]. All of these flaps are known to mostly give good esthetic results. However, esthetic outcome may not be satisfying in all cases. Most adverse effects after repair of defects in the face by flaps are mild. They include persistent scar erythema, pincushioning, and development of hypertrophic and widened scars [1, 5–11].

Current therapies for flap revision after unsatisfactory esthetic results include photothermolysis, laser resurfacing, liposuction, injections with corticosteroids, and surgery [1, 8, 10, 12]. These treatment modalities are invasive, while studies evaluating long-term efficacy of these methods on flaps are lacking. Reliable conservative therapy with long-term stable result is the hiatus in current clinical practice.

Two non-surgical procedures that have been the cornerstone in treatment for hypertrophic and keloid scars for many years are pressure garment therapy and therapy with silicones [13–16].

We hypothesize that combination therapy of silicones and pressure could lead to reducing edema and rigidity as well as cause flattening of the facial flap, as an effect of applying mechanical pressure. Additionally, combination therapy could have scar enhancing and maturation accelerating properties, as effects of both silicones and pressure. In this way, silicone and pressure therapy could act in synergetic fashion to improve esthetic outcome of thickened facial flaps with unsatisfying scars.

In order to incorporate pressure and silicone therapy, we believe that a specialized pressure mask with a silicone layer as inner lining can improve pliability and color and reduce thickness, edema, and irregularities of flaps exposing these qualities. In this study, the efficacy of a unique transparent face mask containing silicone sheets on the esthetic outcome of postsurgical facial flaps is assessed.

## Materials and methods

### Design

In this retrospective study conducted between July 2012 and September 2015, 21 patients were assigned to facial pressure mask therapy with silicones after they underwent flap surgery. Patient characteristics and follow-up information can be seen in Table 1.

**Fig. 1:**
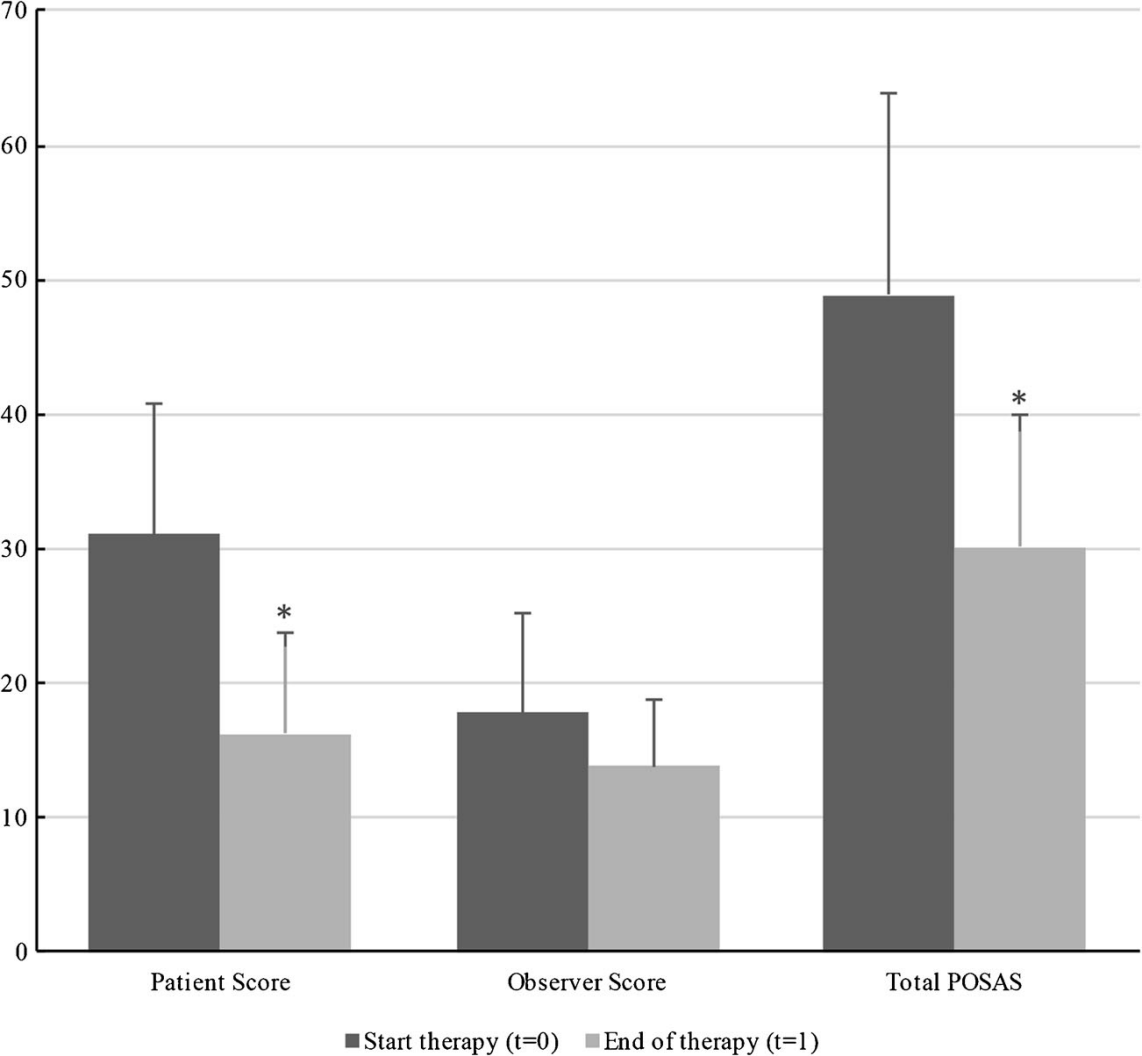
Mean patient, observer, and total POSAS scores are shown at baseline and end of therapy. Statistically significant differences (*P* < 0.05) between means are marked by an *asterisk*

**Fig. 2:**
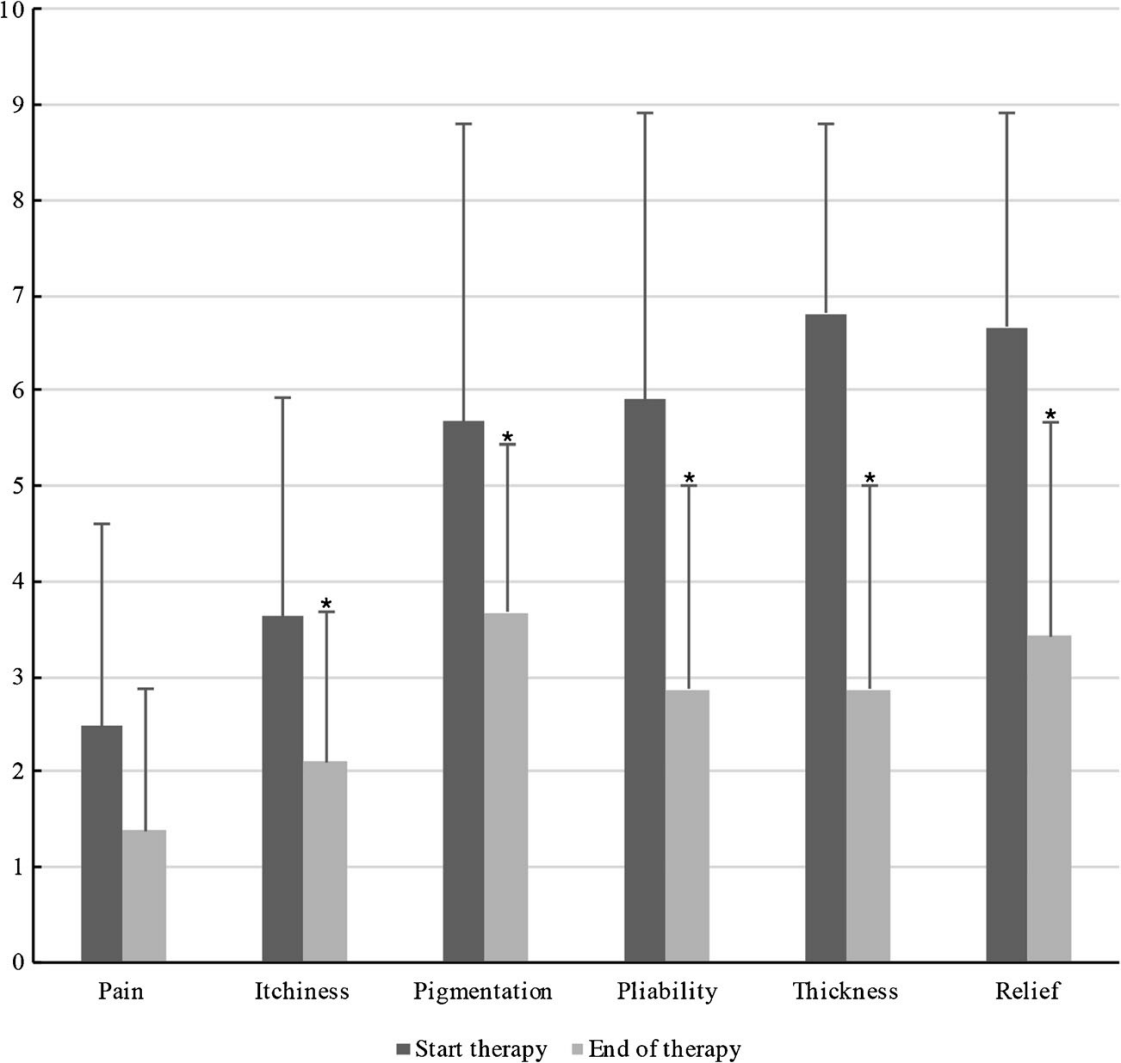
Components of the patient score as part of the total POSAS score are displayed at start and end of therapy. Statistically significant differences (*P* < 0.05) between means are marked by an *asterisk*

**Fig. 3:**
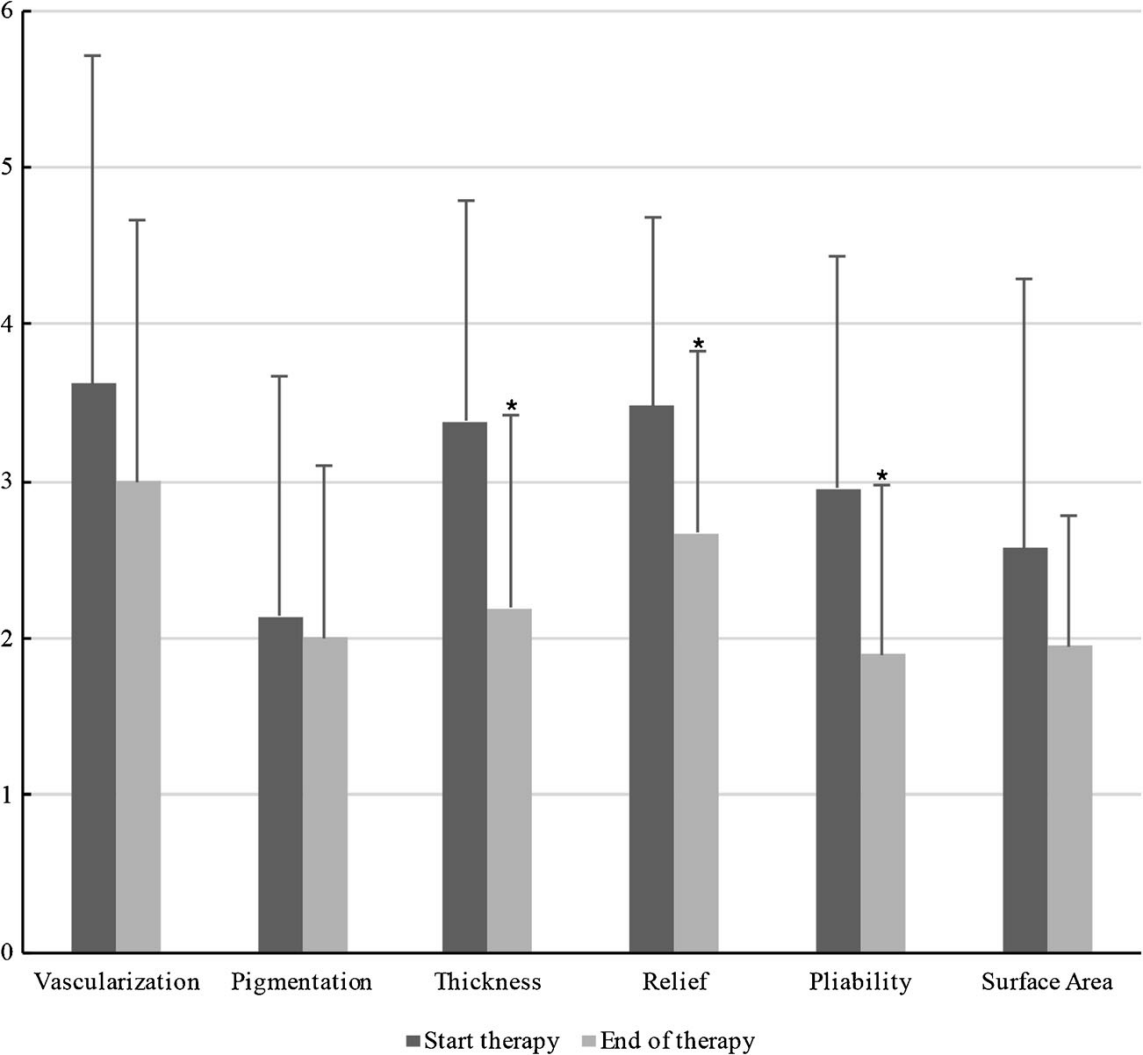
Components of the observer score as part of the total POSAS score are displayed at start and end of therapy. Statistically significant differences (*P* < 0.05) between means are marked by an *asterisk*

**Fig. 4:**
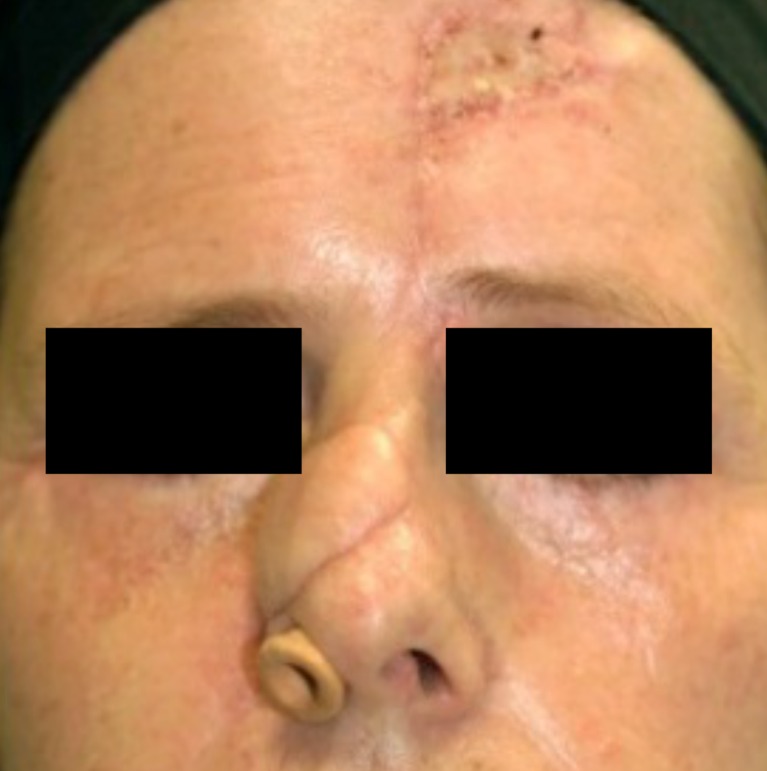
A 50-year old female patient at the start of pressure mask therapy 4 months after surgery

**Fig. 5:**
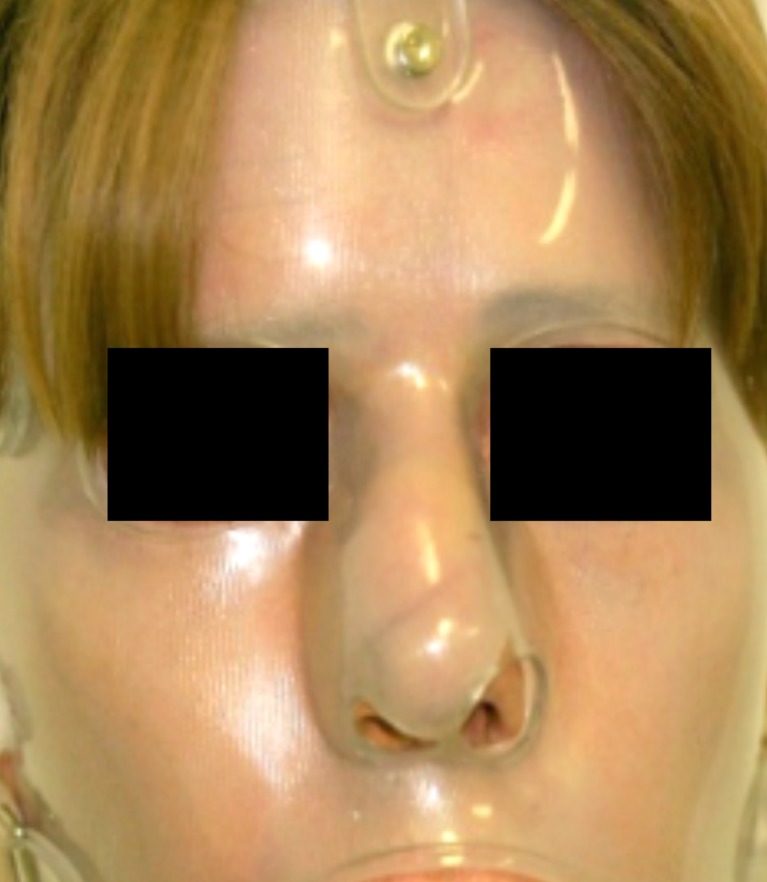
Same female patient with pressure mask applied during therapy

**Fig. 6:**
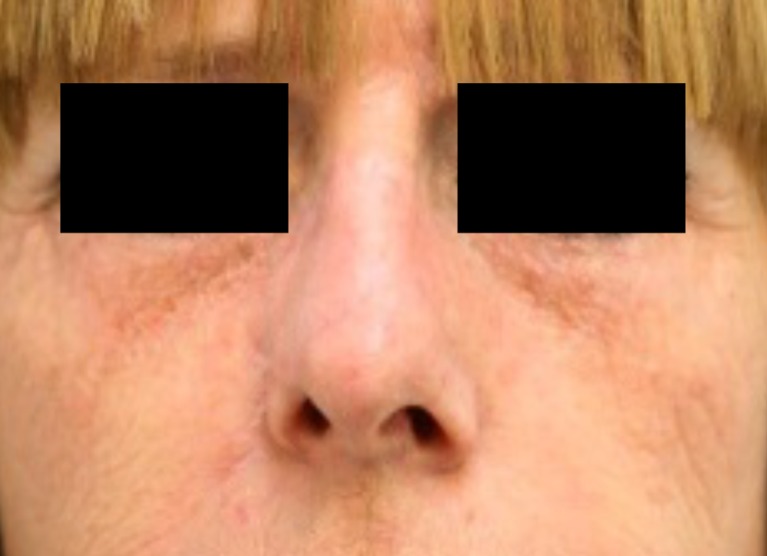
Same female patient at the end of pressure mask therapy 9 months after surgery

**Fig. 7:**
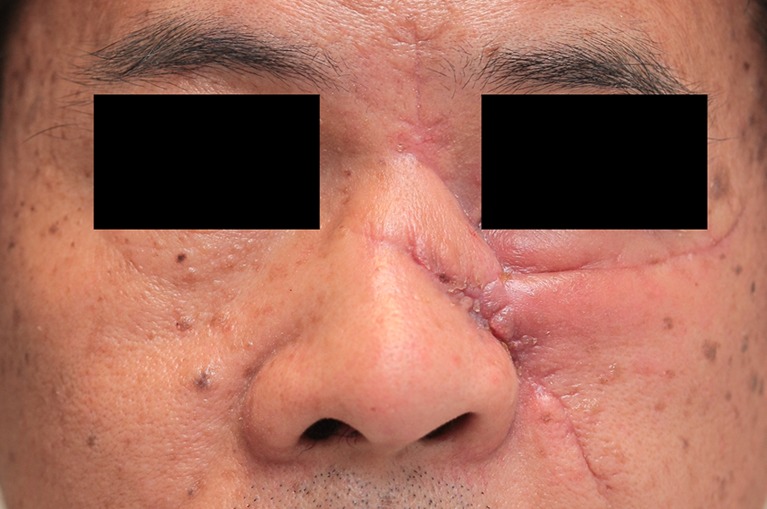
A 48 year-old male patient at the start of pressure mask therapy 3 months after surgery (frontal view)

**Fig. 8:**
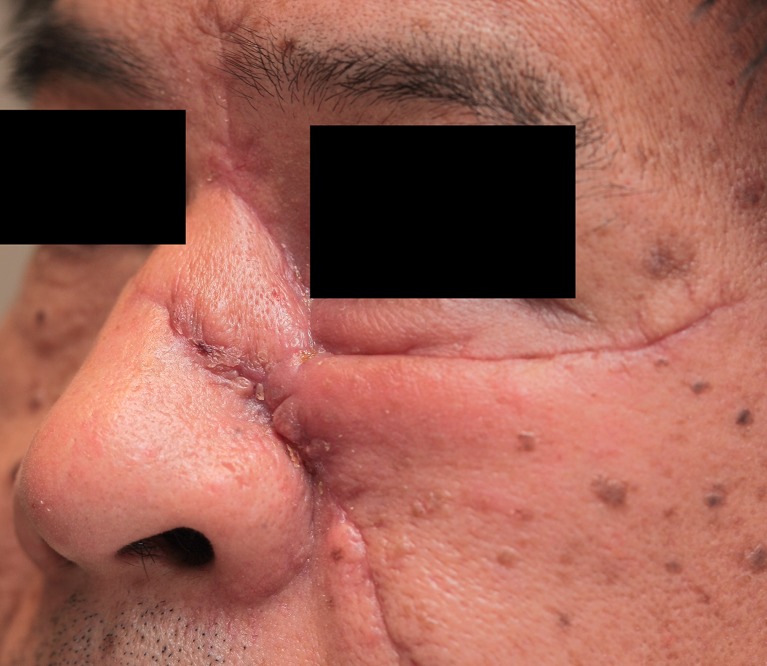
A 48 year-old male patient at the start of pressure mask therapy 3 months after surgery (oblique view)

**Table 1:** Patient characteristics

Age at start therapy (years)	
<40	2
40–49	3
50–59	5
>60	11
Mean age (years)	57
Min	34
Max	80
Gender	
Male	9
Female	12
Follow-up time (weeks)	
10–20	5
20–40	6
40–60	3
60–80	3
80–100	3
>100	1
Mean duration of treatment (weeks)	46
Min	11
Max	112

In order to improve the facial skin functionally and esthetically at the autologous transplantation site (the flap) after surgery, treatment with a transparent polycarbonate facial pressure mask with a silicone layer as inner lining was applied. The different types of surgical flaps used and the reason for surgery are documented in Tables 2 and 3, respectively.

**Table 2:** Surgical flaps used

	No.
Abbe flap	1
Bilobed flap	3
Forehead flap	7
Glabella flap	2
Limberg flap	1
Transposition flap	3
Rotation flap	1
Z-plasty	3

**Table 3:** Reason for flap surgery

	No.
Reconstruction of the face after removal of	
Basal cell carcinoma	13
Melanoma	2
Radical scar excision	3
Sarcoma	1
Squamous cell carcinoma	2

### Patients

Eligible patients were men or women who had received facial flap surgery. Only patients in whom the flap clearly protruded from the normal skin, even after thinning of the flap, were included. Only patients that did not receive any prior therapy for their facial flap were included. If these patients were physically and mentally able and motivated to wear a polycarbonate facial pressure mask for at least 12 h a day, they were suitable and assigned for facial mask therapy. Therapy started when the operated skin passed into the maturation phase of wound healing. Therefore, therapy started between 1 and 3 months after flap surgery took place. The study conformed to good clinical practice guidelines and followed the recommendations of the Declaration of Helsinki. The protocol was approved by the local ethics committee.

### Procedures

From July 2012 to September 2015, 21 patients were assigned to therapy with a transparent polycarbonate facial mask with a silicone layer inside. The mask was fully custom fabricated by an experienced prosthetist.

In order to apply the required pressure to the flap underneath the mask, Velcro straps were attached to the polycarbonate outside of the mask. Targeted pressure was 20 mmHg. Pressure under the mask was measured by means of an aerial pressure sensor and pump.

Patients were advised and insisted to wear the mask as long as they possibly could endure, with the objective to wear the mask at least 12 h every day. When an adequate and satisfying esthetic result was reached, patient and doctor mutually decided to stop therapy. Compliance was evaluated, and if patients were incompliant, mask therapy was stopped. Therapy was also stopped if patients reported a high level of discomfort. All included patients of the current study completed the therapy. The mean duration of the therapy was 46 (11–112) weeks. During this period, patients returned for follow-up every 3 to 4 months at the outpatient clinic. The flaps were assessed at the scar clinic by a team of experts in scar treatment and management including a senior plastic surgeon, a resident plastic surgeon, a prosthetist, and a physiotherapist. At each visit, POSAS forms were filled out, and photographs were taken. Facial mask pressure was monitored on each visit, and when necessary, adjustments of the mask were performed by the prosthetist.

### Assessment of flap

Because no objective and validated tool for assessment of thickened facial flaps exists, we used the previously validated Patient and Observer Scar Assessment Scale (POSAS) for evaluating facial skin and scars at the transposition site [17]. At most 2 weeks prior to fabrication of the mask, the facial skin and scars were first assessed. The flap was rated numerically on a 10-step scale by both the patient and doctor on six items. The Observer Scale rates vascularity, pigmentation, thickness, relief, pliability, and surface area. The Patient Scale consists of pain, itchiness, color, pliability, thickness, and relief. Patients were informed to assess the facial reconstruction as a whole (both flap and scar together) not solely the scar, on the six components of the Patient Scale.

One of the reasons POSAS was chosen for flap evaluation is because it is the only scar assessment tool to include a component for patients to fill out. Furthermore, we chose POSAS because of its distinctive feature of reflecting subjective symptoms like pain and itchiness and because of its usefulness for everyday practice [18–20].

On each visit, an expert and the patient independently filled in a POSAS form in order to assess the transposition site.

### Data analysis

POSAS scores are presented as means with standard deviations. Those scores were compared with the use of one-way ANOVA for significance in means. Two-tailed values of *P* < 0.05 were accepted as statistically significant. All analyses were performed using the statistical software program SPSS 22.0.

## Results

### Outcome mean patient, observer, and total POSAS score

All flap sites were evaluated prior to or on the day the mask therapy started by means of POSAS scores. POSAS scores at baseline and at the end of therapy were compared by means of one-way ANOVA.

Figure 1 and Table 4 show that mean POSAS scores decreased significantly (*P* < 0.05) between baseline and end of therapy, with a total of 18.72 points.

**Fig. 9:**
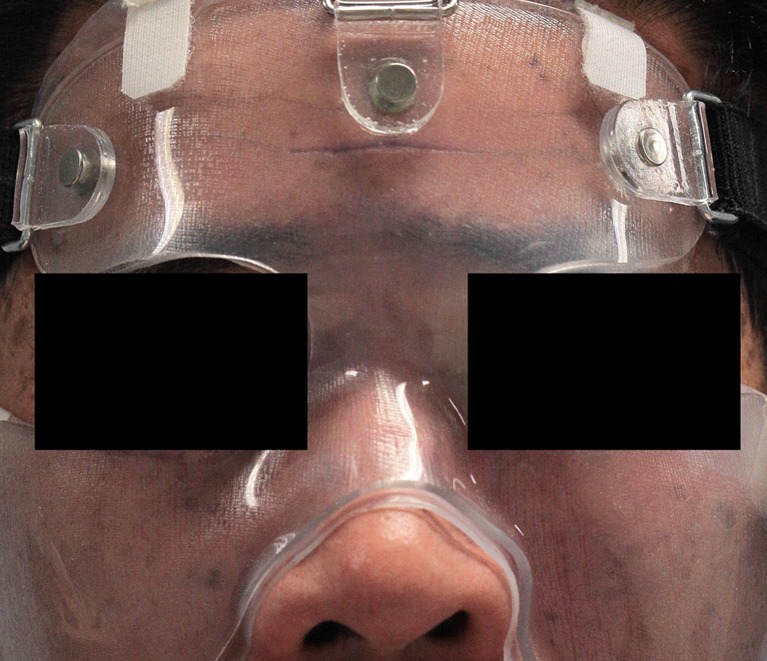
Same patient with pressure mask applied during therapy

**Table 4:** Mean total POSAS scores

	Start therapy	SD	End of therapy	SD	*P* value
Total POSAS	48.86	14.97	30.14	9.82	<0.001

Patient scores also decreased significantly (*P* < 0.05) with a mean total of 14.81 points, between baseline and end of therapy (Table 5).

**Table 5:** Mean patient scar scores

Overall patient score					
	Start therapy	SD	End of therapy	SD	*P* value
Pain	2.48	2.21	1.38	1.32	0.060
Itchiness	3.62	2.42	2.10	1.81	0.026
Pigmentation	5.67	2.99	3.67	1.74	0.012
Pliability	5.90	2.64	2.86	2.01	<0.001
Thickness	6.81	1.81	2.86	2.01	<0.001
Relief	6.67	2.13	3.43	2.04	<0.001
Patient score	31.10	9.76	16.29	7.43	<0.001

Observer scores showed a mean reduction of 3.90 points between baseline and end of therapy. However, this reduction was not statistically significant (Table 6).

**Table 6:** Mean observer scar scores

Overall observer score					
	Start therapy	SD	End of therapy	SD	*P* value
Vascularization	3.62	1.88	3.00	1.55	0.252
Pigmentation	2.14	1.42	2.00	1.05	0.713
Thickness	3.38	1.40	2.19	1.08	0.004
Relief	3.48	1.20	2.67	1.16	0.035
Pliability	2.95	1.43	1.90	1.09	0.011
Surface area	2.57	1.63	1.95	0.87	0.132
Observer score	17.76	7.38	13.86	4.99	0.051

### Patient compliance

At every follow-up visit, patients were asked how long they wore the mask. In general, patients declared they wore the mask for a mean time of 10 to 12 h a day.

### Patient scores

Table 5 and Fig. 2 show the six characteristics of the patient score (pain, itchiness, pigmentation, pliability, thickness, and relief) at start and end of therapy. All patient score components showed a decrease after baseline. The largest decrease was observed in thickness, with a mean reduction of 3.95 points at the end of therapy. Itchiness, pigmentation, pliability, thickness, and relief showed a statistically significant reduction (*P* < 0.05) between baseline and end of therapy.

**Fig. 10:**
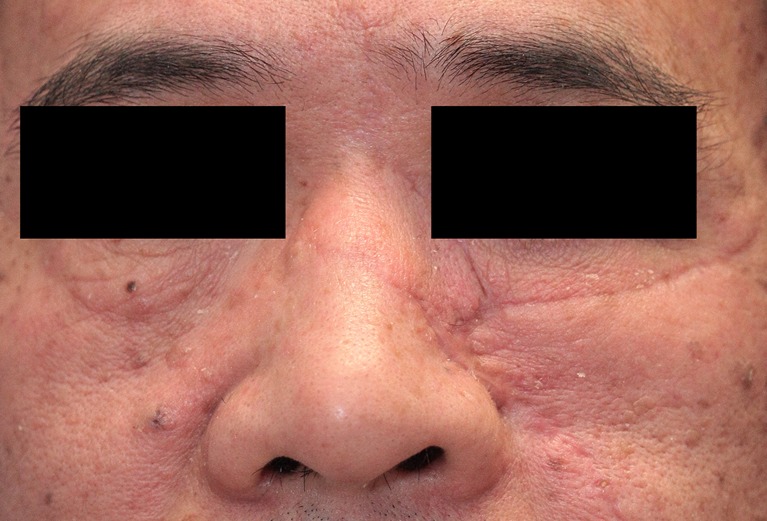
Same male patient at the end of pressure mask therapy 15 months after surgery (frontal view)

### Observer scores

**Fig. 11:**
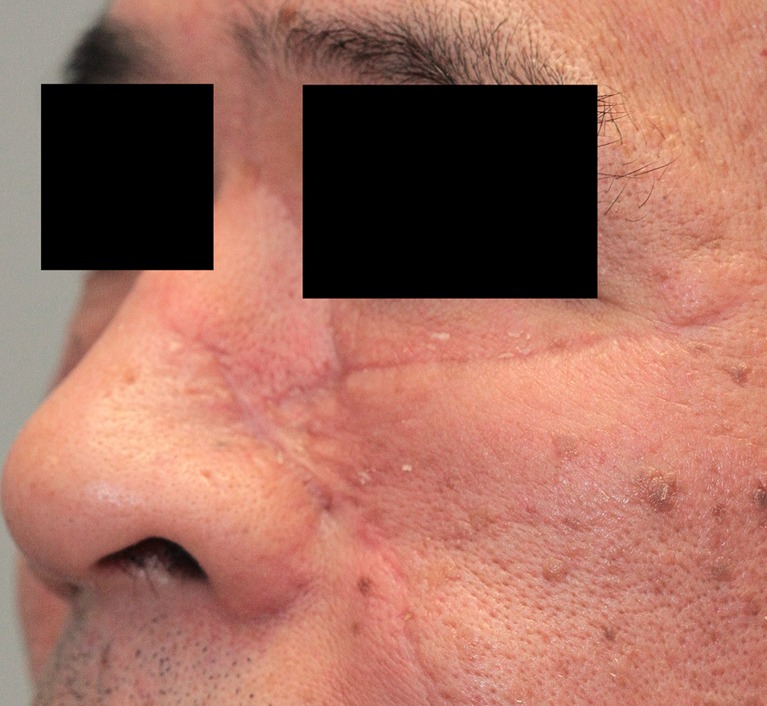
Same male patient at the end of pressure mask therapy 15 months after surgery (oblique view)

Table 6 and Fig. 3 show the six characteristics of the observer score (vascularization, pigmentation, thickness, relief, pliability, and surface area) at start and end of therapy. Corresponding to the patient scores, all components of the observer score decreased after start of therapy. The largest decrease in observer score was seen in flap thickness, with a mean reduction of 1.19 points between baseline and end of therapy. Pliability, thickness, and relief were the observer score components that showed statistically significant reduction (*P* < 0.05). Representative cases are depicted in Figs. 4, 5, 6, 7, 8, 9, 10, and 11.

## Discussion

### Pressure therapy

Mechanical compression by means of pressure garment therapy is a reputable modality to diminish collagen synthesis by reducing blood flow. Another hypothesis for the action of pressure therapy is a decrease in blood flow that causes hypoxia, resulting in fibroblast degeneration and loosening of collagen fibrils [21, 22]. Pressure garment therapy has also proven to result in significant improvement in scar erythema and thickness in postburn hypertrophic scars [23].

Since the 1980s roughly, various types of facial topical therapies have been described [24, 25]. The transparent face mask or transparent face orthosis is a well-known and effective modality in the management of burn-related hypertrophic scars, as it has proven to reduce hypertrophic scars significantly [26]. For the fabrication process of the mask, different techniques exist, among the formation of both manual and laser-generated molds [24, 27].

When pressure therapy is applied to flaps in extremities, it reduces edema, and it helps to reconstruct and reshape a defect after flap surgery [28]. Further evidence for pressure therapy in reshaping and correcting flaps can mainly be addressed as anecdotal.

### Silicone therapy

Silicone therapy for scars has become standard practice among plastic surgeons, as there is good clinical evidence of the efficacy [29, 30]. One of the mechanisms to occur after application of silicones to the skin is an increase of hydration. As an effect of increased hydration, capillary activity could decrease, thereby reducing local collagen deposition [31]. The clinical effects of increased hydration include improvement of pruritus, pain, pliability, and decrease of edema [32, 33].

### Combination therapy

However, the combined effect of pressure therapy and silicones is less studied. A randomized controlled trial (RCT) demonstrated that silicones combined with pressure therapy were associated with significant improvement in pliability, thickness, and vascularity of 38 hypertrophic burn scars [34]. A smaller pilot RCT showed inconclusive evidence on the potential beneficial effect of combination therapy on 30 hypertrophic burn scars [35]. A larger RCT demonstrated combined therapy to be effective in improving thickness of hypertrophic postburn scars, compared to silicone and pressure therapy separately [36].

In this study, we have shown that specialized facial mask therapy significantly improves esthetic outcome after facial flap surgery. Mean total POSAS scores showed a significant decrease between baseline and end of therapy (Table 4, Fig. 1), with mean therapy duration of 46 weeks (Table 1).

Our results show patients’ opinion about their facial reconstruction improved the most, with a significant improvement (*P* < 0.05) in itchiness, pliability, pigmentation, thickness, and relief (Table 5, Fig. 2).

Overall, observer POSAS scores did not show significant reduction over time (*P* = 0.051). However, thickness, relief, and pliability, as part of the observer score, did reduce significantly (Table 6).

To our knowledge, extensive and long-term studies about esthetic outcome after facial flap surgery are lacking. Also, the effect of a facial pressure mask for improvement in esthetic outcome for flaps has not been documented earlier. A limited amount of methods for esthetic refinement after flap surgery is available in current clinical practice. Yet, intraoperative intradermal injections of methylprednisolone can reduce flap edema, according to an animal study [37]. Other methods for esthetic refinements after facial flap surgery include photothermolysis and laser resurfacing.

In accordance with our results, we believe that our specialized pressure mask could be an effective modality to avoid surgical debulking or thinning of flaps, with small risk of complications and adverse effects in contrast to surgery.

### Strengths and limitations

This is the first clinical cohort study to assess the usefulness of a facial pressure mask with silicones for enhancement after facial flap surgery. There were some limitations of this study. In the absence of a flap assessment scale, the validated and well-known clinical scar assessment tool POSAS is the only instrument for flap assessment used in this study. Other qualitative methods for judging flaps are lacking in this study. Preferably, the current study would contain a larger study population and a control group. However, the vast majority of patients appear to have good esthetic results after facial flap surgery, causing the remaining eligible patient group to be of small size. Additionally, the burden of wearing a pressure mask for at least 12 h a day should not be underestimated. The strength of this study is that it shows clearly that patients who wore a pressure mask had strong esthetical improvement of the facial reconstruction site.

### Further research

In order to assess esthetic outcomes after flap reconstruction, more extensive and precise, 3D digital flap volume measurements, as well as continuous pressure measurements underneath the mask, would be of great value. Ideally, further studies would contain a control group, considering that information about the natural course of thickened, hypertrophic, and unesthetic facial flaps over time is lacking.

A major goal of this study was the improvement in overall esthetic outcome: restoration of flap skin close to normal skin. In this study, patients showed a clinical significant result in esthetic improvement of facial flaps.

## Conclusion

In this study, our aim was to assess the efficacy of a specialized facial pressure mask on the esthetic outcome of facial flaps, since no other study examined the effect of facial pressure therapy with silicones on flap enhancement over a reasonable amount of time. Our retrospective study showed that a transparent facial pressure mask with silicones results in noticeable flap improvement with a long-lasting result, particularly in our patients’ view.
